# Supplementary material for: Pulmonary Edema in COVID-19 Treated with Furosemide and Negative Fluid Balance (NEGBAL): A Different and Promising Approach
Source: J Clin Med. 2021 Nov 28;10(23):5599. doi: 10.3390/jcm10235599 (PMC8658626; doi:10.3390/jcm10235599)
Supplement: Supplementary file 1 [file jcm-10-05599-s001.zip › jcm-1450547-supplementary.pdf]

Supplemental Material

Pulmonary edema in COVID-19 treated with furosemide and negative fluid balance (NEGBAL): a different and promising approach

Jose L. Francisco Santos, MD[1]; Patricio Zanardi, MD[1]; Veronica Alo, MD[1]; Marcelo Rodriguez, MD[2]; Federico Magdaleno, MD[3]; Virginia De Langhe, MD[3]; Vanina Dos Santos, MD[1]; Giuliana Murialdo, MD[1]; Andrea Villoldo, MD[1]; Micaela Coria, MD[1]; Diego Quiros, MD[4]; Claudio Milicchio, MD[4]; Eduardo García Saiz, MD[4].

[1]Intensive Care Unit, Clinica Colon, Mar del Plata, Buenos Aires, Argentina.

[2]Cardiology Service, Clinica Colon, Mar del Plata, Buenos Aires, Argentina.

[3]Diagnostic Imaging Service, Hospital Privado del Sur. Bahía Blanca, Buenos Aires, Argentina.

[4]Diagnostic Imaging Service. Clinica Colon, Mar del Plata, Buenos Aires, Argentina.

1. **Supplement 1: Supplementary Tables**
2. **Supplement 2: Supplementary Figures**
3. **Supplement 3: Supplemental Statistics**

Supplement 1

Supplementary Tables

This supplement with tables and figures has been provided by the authors to give readers additional information about their work.

Table S1. Daily Negative Fluid Balance in milliliters per day. Accumulate in milliliters total days of Negative Fluid Balance (NEGBAL).

CASE	*Day 1*	*Day 2*	*Day 3*	*Day 4*	*Day 5*	*Day 6*	*Day 7*	*Day 8*	*Accumulated*
**1**	-1250	-700	-1000	155	-630				-3425
**2**	-250	-300	-2400	-3500	-2800	-1500	-500		-11250
**3**	-1950	-1700	-1400	-1300	-1300	-1150			-8800
**4**	-1960	-1600	-1600	-784	-993				-6937
**5**	-1400	-250	-1200	-1200	-800	-1500	-1000	-2000	-9350
**6**	-1000	-1500	250	-1400	-1300	-2100	-1300	-1900	-10250
**7**	-600	-1000	-900	-700	-350	-450	-300	-300	-4600
**8**	-1500	-2100	-1100	-1500	-1300	-1450	-800	-1130	-10880
**9**	-1600	-2400	-1200	-1600					-6800
**10**	-320	-450	-960	-1100	-2000	-1500	-1300		-7630
**11**	-125	-422	-1118	-992	-200	-790			-3647
**12**	-1300	-2600	-880	-1475	-820	-994	-1370		-9439
**13**	-850	-1000	-460	-1238	-950	-1640	-1700		-7838
**14**	-1400	-2900	-900	-2258					-7458
**15**	-620	183	-1570	-1500	-1200	-308	-630		-5645
**16**	-829	-1914	-1377	-1370					-5490
**17**	-1795	-905	-522	-316	440	-200			-3298
**18**	-462	-52	-2280	-1870	-1000	-2200	-360	-936	-9160
**19**	-1880	-2440	-788	-1018	-1200	-2620	-925	-730	-11601
**20**	-1600	-1080	-1591	-1324	-1285	-1244	-1124		-9248

Table S2. Heart Rate daily evolution.

CASE	Admission	Day 1	Day 2	Day 3	Day 4	Day 5	Day 6	Day 7
**1**	68	63	75	53	59	87	82	58
**2**	63	88	98	90	86	88	76	80
**3**	55	65	54	67	66	70	70	70
**4**	68	71	73	80	68	74	77	-
**5**	86	108	98	85	84	80	76	71
**6**	72	70	76	82	80	78	80	82
**7**	85	61	72	67	71	72	65	80
**8**	80	79	83	69	73	54	58	60
**9**	80	80	91	59	78	-	-	-
**10**	108	75	80	85	90	90	90	85
**11**	88	64	60	60	68	86	78	68
**12**	84	67	65	62	65	81	60	60
**13**	84	88	74	60	66	64	57	60
**14**	91	78	76	78	76	73	-	-
**15**	81	68	83	71	-	65	105	66
**16**	95	89	74	60	79	-	-	-
**17**	64	71	95	95	95	70	90	-
**18**	74	81	56	70	62	67	75	-
**19**	95	72	80	69	78	65	67	66
**20**	105	99	87	77	100	68	69	80

Table S3. Temperature (ºC) daily evolution.

CASE	Admission	Day 1	Day 2	Day 3	Day 4	Day 5	Day 6	Day 7
**1**	36.50	35.00	35.90	35.20	35.90	36.80	36.50	35.00
**2**	36.00	36.50	36.00	36.70	35.50	37.00	36.00	36.10
**3**	36.00	36.00	36.00	36.00	36.00	36.30	36.50	36.60
**4**	37.10	36.00	35.70	35.60	36.60	36.20	35.60	-
**5**	37.60	37.10	36.80	36.00	36.50	36.50	36.70	36.60
**6**	36.70	35.40	36.00	36.10	36.80	36.00	36.50	36.10
**7**	36.20	35.20	36.00	36.20	36.00	36.10	36.30	35.70
**8**	36.00	36.10	36.10	35.80	35.70	36.10	35.60	35.50
**9**	36.00	36.00	35.80	36.10	35.60	-	-	-
**10**	35.30	35.50	36.00	36.00	36.00	36.00	36.00	36.00
CASE	***Admission***	***Day 1***	***Day 2***	***Day 3***	***Day 4***	***Day 5***	***Day 6***	***Day 7***

**11**	37.00	36.00	35.60	36.50	36.10	35.80	35.50	35.50
**12**	39.00	37.00	37.40	36.20	35.30	35.80	35.50	35.60
**13**	36.20	36.00	36.00	35.80	35.50	36.00	36.00	36.10
**14**	36.00	36.00	35.60	35.70	35.00	35.30		-
**15**	36.90	36.60	36.00	36.10		35.90	35.90	-
**16**	36.00	36.70	37.20	36.00	-	-	-	-
**17**	35.00	35.90	35.60	35.00	35.00	35.00	-	-
**18**	39.00	39.10	36.00	36.00	36.60	36.50	36.80	-
**19**	36.90	36.00	36.20	35.50	35.00	36.00	35.50	36.10
**20**	37.90	36.20	35.40	36.00	36.00	36.00	35.60	35.40

Table S4. Serum Creatinine Levels at ICU Admission and Discharge

CASE	***Admission***	***Discharge***
**1**	13	-
**2**	6	4.4
**3**	8.2	7.5
**4**	6.7	11.2
**5**	8.2	8.9
**6**	6.9	7.3
**7**	7.1	8.5
**8**	12.1	9.1
**9**	7.6	9.4
**10**	10	8
**11**	9.1	8.3
**12**	10.3	10.4
**13**	9.4	11
**14**	9	10.1
**15**	11.1	12.3
**16**	10	10.7
**17**	7	-
**18**	9	13.4
**19**	7.1	7.2
**20**	9.5	10.2

ICU: Intensive Care Unit

Table S5. Serum sodium levels at ICU Admission and Day 7		
CASE	***Admission***	***Day 7***
**1**	137.00	-
**2**	141.00	137.00
**3**	142.00	142.00
**4**	136.00	-
**5**	137.00	132.00
**6**	136.00	134.00
**7**	137.00	-
**8**	137.00	134.00
**9**	136.00	-
**10**	132.00	132.00
**11**	138.00	133.00
**12**	138.00	135.00
**13**	136.00	132.00
**14**	135.00	-
**15**	135.00	135.00
**16**	136.00	-
**17**	136.00	141.00
**18**	137.00	-
**19**	136.00	-
**20**	137.00	135.00

ICU: Intensive Care Unit

Table S6. Serum potassium at ICU Admission and Day 7

CASE	***Admission***	***Day 7***
**1**	3.9	-
**2**	3.1	3.8
**3**	4.1	3.8
**4**	4	-
**5**	4.7	5
**6**	4.5	4.4
**7**	4.2	-
**8**	4.3	4.5
**9**	4.1	-
**10**	4.4	3.1
**11**	3.8	3.5
**12**	4.2	3.1
**13**	4.5	3.9
**14**	4.4	-
**15**	3.1	5
**16**	3.6	-
**17**	3.5	4.5
**18**	4.1	-
**19**	4.2	3.5
**20**	3.8	3.8

ICU: Intensive Care Unit

Table S7. CT Score PRE, CT Score ADM, CT Score Day 4 and CT Score Day 8 data.				
CASE	***CT Score PRE***	***CT Score ADM***	***CT Score Day 4***	***CT Score Day 8***
**1**	6	21	14	7
**2**	-	13	8	4
**3**	12	20	3	3
**4**	5	17	1	1
**5**	10	17	17	7
**6**	7	14	10	10
**7**	10	23	23	16
**8**	8	19	5	5
**9**	9	18	12	12
**10**	-	25	25	25
**11**	-	12	2	0
**12**	10	20	10	3
**13**	2	16	9	5
**14**	6	11	3	3
**15**	-	22	18	12
**16**	1	12	10	9
**17**	-	17	12	7
**18**	-	17	17	16
**19**	-	11	6	5
**20**	-	19	8	5
**Mean**	7.16	17.2	10.6	7.75
**Median**	7.5	17	10	6
**Standard deviation**	1.43	1.34	1.62	1.41

Table S8. Superior Vena Cava Diameter (mm) in Computed Tomography (CT).

CASE	CT previous to NEGBAL	CT at NEGBAL onset	CT at day 4	CT at day 8	CT at day 12
**1**	17.1	18.9	-	16.8	-
**2**	-	19.1	16.4	-	15
**3**	18.4	25.2	12.4	-	-
**4**	12.5	16.7	11.4	-	-
**5**	15.1	18.3	17.4	15.9	-
**6**	13.9	20.1	15.5	-	-
**7**	22.7	25.4	26.5	17.2	-
**8**	8.3	20.2	13.2	-	-
**9**	19.2	20.9	19.4	-	-
**10**	-	18.3	19.4	17.4	18.2
**11**	-	12.3	5.5	8.1	-
**12**	12.8	19.5	13.6	12.5	-
**13**	10.7	14.5	10.8	8.1	-
**14**	17.1	14.4	12.2	-	-
**15**	-	15.9	15.1	15	15.6
**16**	9.5	10.4	13.9	12.6	-
**17**	-	17.1	10.8	14.4	-
**18**	-	16.1	15.5	-	-
**19**	-	20	9.1	15.9	-
**20**	-	14.2	10.1	9.5	-

NEGBAL: Negative Fluid Balance approach

Table S9. Cardiac Axis (cm) in Computed Tomography (CT).					
CASE	***Cardiac Axis previous to NEGBAL***	***Cardiac Axis at NEGBAL onset***	***Cardiac Axis at day 4***	***Cardiac Axis at day 8***	***Cardiac Axis at day 12***
**1**	11.7	12.5	-	12.3	-
**2**	-	12.8	10.8	-	11.3
**3**	12.2	14	11.8	-	-
**4**	10.1	11.2	8.3	-	-
**5**	13.7	14.7	14.4	13.5	-
**6**	10.5	11.8	9.3	-	-
**7**	13.1	13.4	13.9	12.7	-
**8**	10.9	13.6	11.4	-	-
**9**	14	15	12.9	-	-
**10**	-	12	10.7	11.9	10.9
**11**	-	11.4	10	10	-
**12**	14.2	15	14.1	14.1	-
**13**	11.7	13.8	13.4	13.1	-
**14**	13.4	12.6	11.4	-	-
**15**	-	11.5	10.6	11.1	10.9
**16**	11	11.1	12	11.7	-
**17**	-	11.7	10.8	12.1	-
**18**	-	12.5	12	-	-
**19**	-	11.3	10.7	10.9	-
**20**	-	14.5	13	12.6	-

NEGBAL: Negative Fluid Balance approach

Table S10. Cardiothoracic Ratio (CTR) in Computed Tomography (CT).

CASE	***CTR previous to NEGBAL***	***CTR at NEGBAL onset***	***CTR at day 4***	***CTR at day 8***	***CTR at day 12***
**1**	0.45	0.47	-	0.45	-
**2**	-	0.56	0.48	-	0.51
**3**	0.45	0.54	0.45	-	-
**4**	0.39	0.44	0.34	-	-
**5**	0.44	0.47	0.47	0.44	-
**6**	0.40	0.49	0.37	-	-
**7**	0.55	0.57	0.60	0.55	-
**8**	0.37	0.52	0.41	-	-
**9**	0.47	0.51	0.44	-	-
**10**	-	0.47	0.42	0.47	0.43
**11**	-	0.50	0.43	0.44	-
**12**	0.52	0.57	0.53	0.51	-
**13**	0.42	0.49	0.48	0.47	-
**14**	0.48	0.46	0.41	-	-
**15**	-	0.44	0.40	0.42	0.41
**16**	0.42	0.44	0.48	0.46	-
**17**	-	0.53	0.48	0.55	-
**18**	-	0.51	0.48	-	-
**19**	-	0.47	0.45	0.45	-
**20**	-	0.54	0.49	0.47	-

NEGBAL: Negative Fluid Balance approach

Table S11. Furosemide in milligrams per hour data daily evolution.

CASE	*Day 1*	*Day 2*	*Day 3*	*Day 4*	*Day 5*	*Day 6*	*Day 7*
**1**	3.30	3.30	3.30	2.50	2.50	2.50	2.50
**2**	1.70	1.70	1.70	1.70	0.40	-	-
**3**	3.30	3.30	3.30	1.60	2.50	-	-
**4**	5.00	9.10	910	5.00	2.50	-	-
**5**	2.50	0.80	1.60	1.60	2.50	0.80	
**6**	2.50	0.80	0.80	0.80	0.80	0.40	0.40
**7**	1.70	2.50	250	3.30	3.30	3.30	3.30
**8**	1.70	2.50	2.50	2.50	2.50	-	-
**9**	1.70	2.50	2.50	2.50	2.50	-	-
**10**	5.00	5.00	8.00	8.00	8.00	4.00	-
**11**	5.00	4.10	9.10	9.10	14.10	14.10	9.10
**12**	5.00	4.10	4.10	7.50	7.50	4.10	2.50
**13**	3.30	9.10	4.10	10.00	4.10	2.50	-
**14**	1.70	2.50	2.50	2.50	2.50	-	-
**15**	1.70	1.70	2.50	2.50	5.00	2.50	-
**16**	7.50	7.50	10.00	5.00	-	-	
**17**	4.30	10.00	10.00	6.00	10.00	-	-
**18**	2.50	2.50	0.80	0.00	2.50	2.50	-
**19**	9.00	9.00	9.00	12.00	12.00	12.00	12.00
**20**	3.30	3.00	3.00	6.00	6.00	9.00	9.00
**Mean**	3.58	4.25	4.52	4.5	4.8	4.81	5.54
**Median**	3.3	3.15	3.15	2.9	2.5	2.9	3.3
**Standard deviation**	2.03	2.98	3.28	3.356	3.83	4.43	4.4

Supplement 2

Supplementary Figures

Figure S1. What is hidden behind this evolution COVID 19?

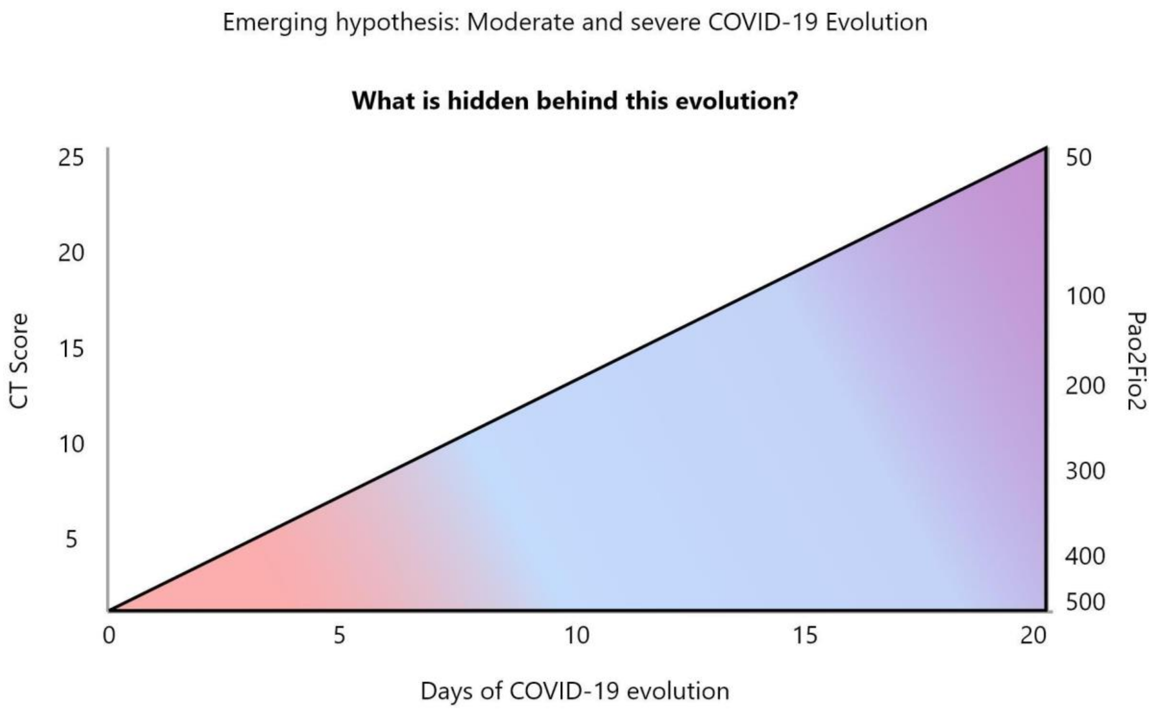

Figure S2-. Emerging hypothesis: First HIT

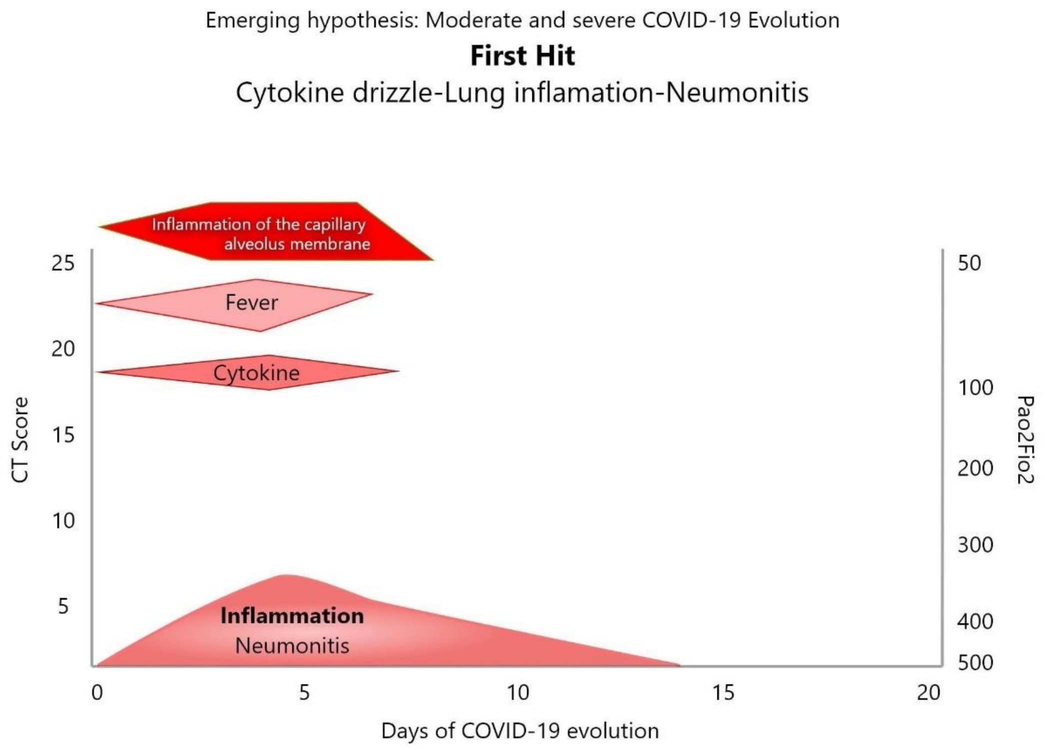

Figure S3-. Emerging hypothesis: Second HIT.

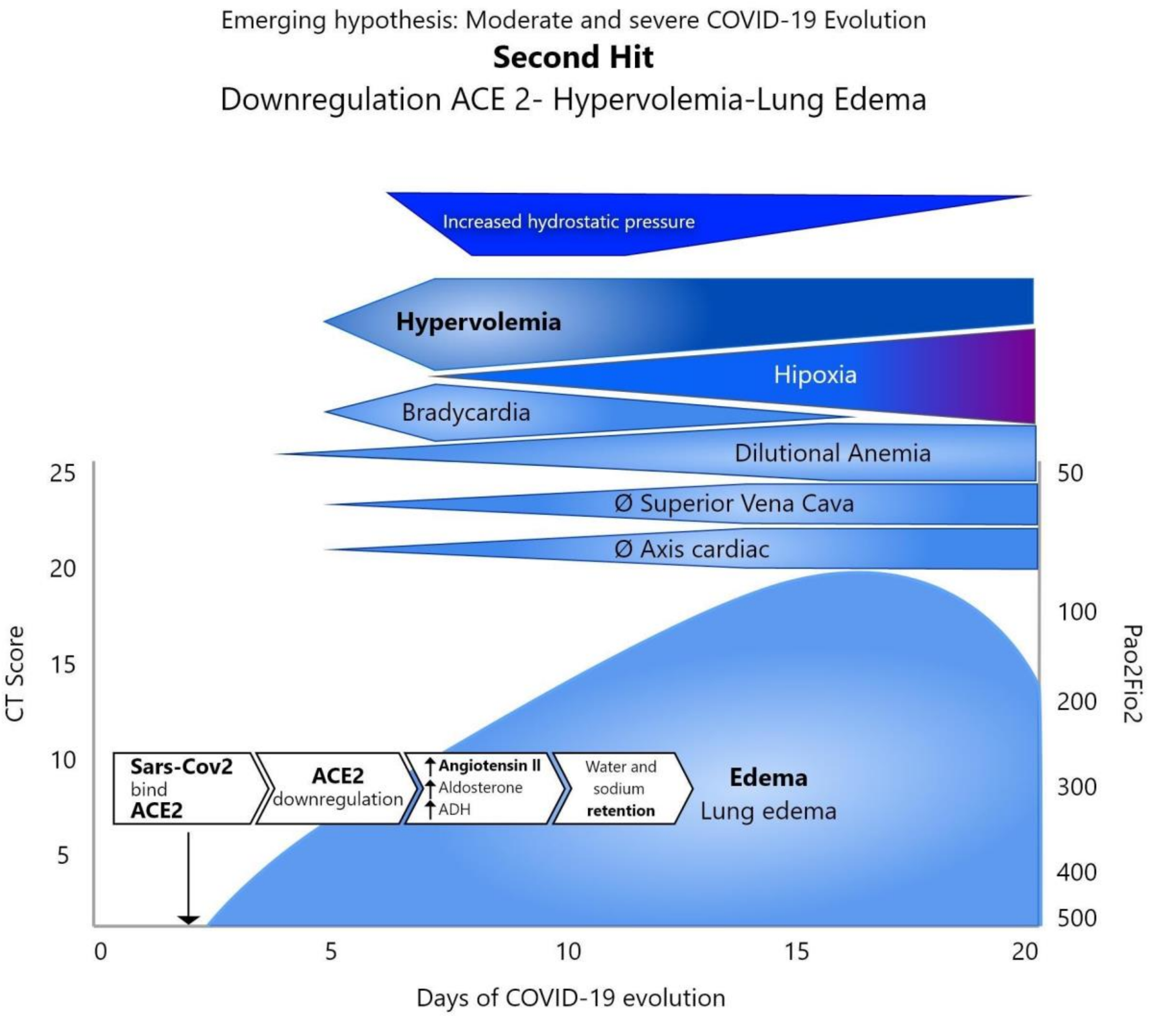

Figure S4-. Emerging hypothesis: Third HIT.

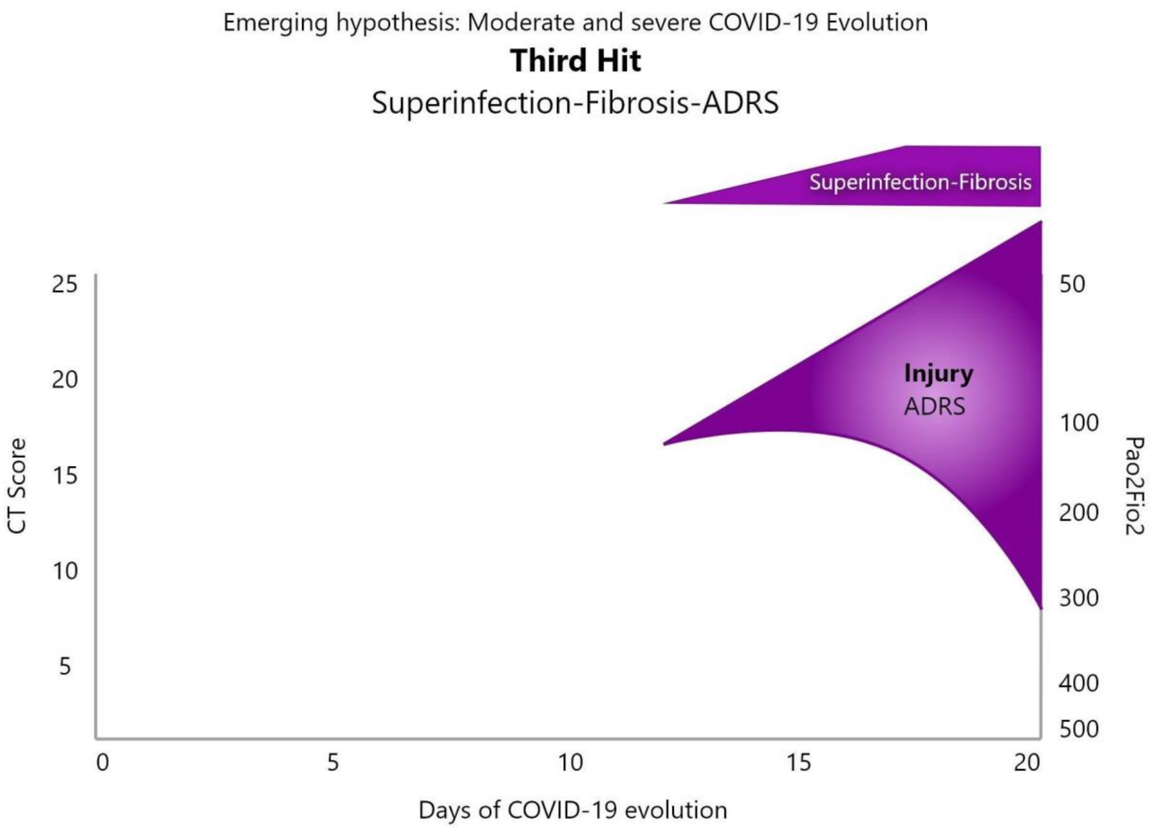

Figure S5-. Emerging hypothesis: COVID 19: Three different and successive HITS.

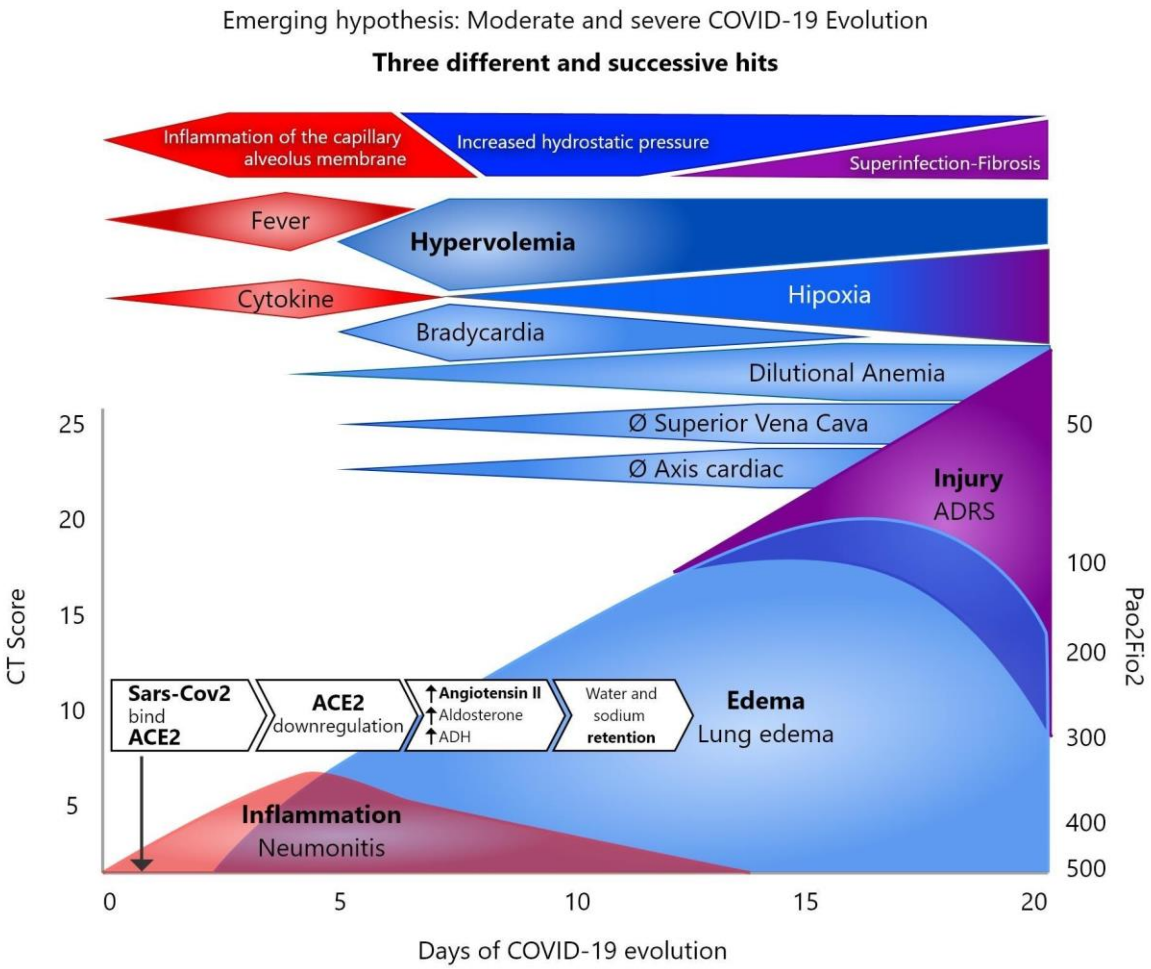

Figure S6: NEGBAL approach for emerging hypothesis.

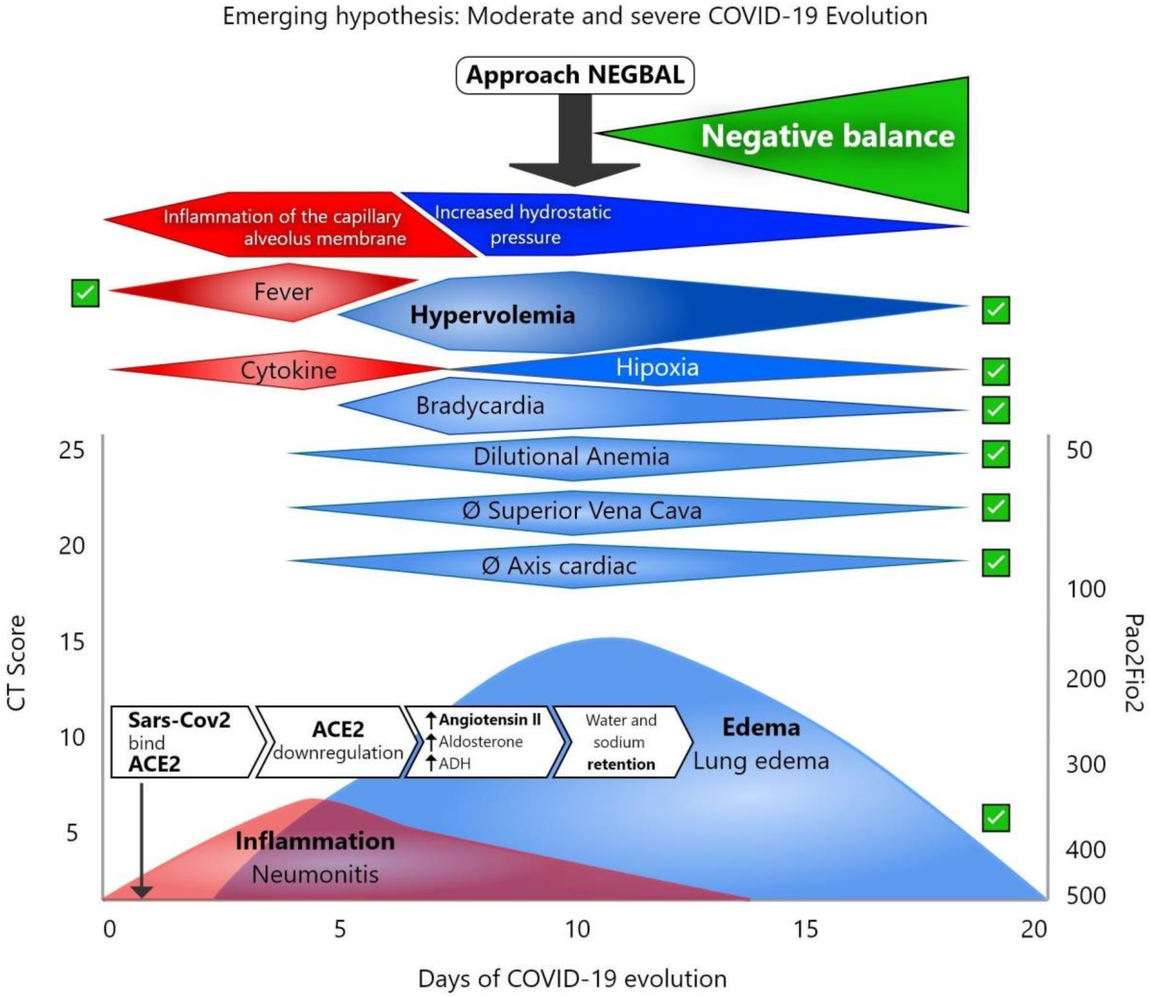

Supplement 3

Supplemental Statistics

Statistical report:
COVID-19 treatment with NEGBAL protocol

Dr. Hernán A. Vivas
Centro Marplatense de Investigaciones Matemáticas (UNMdP - CIC)

Contents

1. **Data source and description**
2. **Statistical model**
3. **Results**
4. **Appendix 1: Security variables**
5. **Appendix 2: Thoracic index and furosemide**

1. Data source and description

We analyzed data from 20 patients treated for COVID-19 disease following the NEGBAL (Negative Fluid Balance approach) protocol for four to eight days[1]. The data consisted of: a) the negative fluid balance for each day and each patient and the cumulative negative fluid balance (total NEGBAL) of each patient; b) daily measurements of the $P_{aO}2$ /$F_{IO}2$ ratio (PAFI) for each patient throughout the treatment, in particular from the day of admission (PAFI ADM), the fourth and eight days of treatment (PAFI D04 and PAFI D08) and the day of discharge (PAFI DIS); c) hematocrit (HCT) measurements for the days of admission (HCT ADM) and discharge (HCT DIS) of all the patients, and previous hematocrit measurements from clinical records for some of the patients (HCT PRE); d) Computed Tomography Score (CT SCORE) measurements from the day of admission (CT SCORE ADM), the fourth day of treatment (CT SCORE D04) and the eight day of treatment (CT SCORE D08) and previous CT score measurements from clinical records for some of the patients (CT SCORE PRE); e) CT measurements of the diameter superior vena cava (Ø SVC) from the day of admission (CT Ø SVC ADM), the fourth day of treatment (CT Ø SVC D04) and the day of discharge of each patient (CT Ø SVC DIS) and previous CT measurements of diameter of superior vena cava from clinical records for some of the patients (CT Ø SVC PRE); f) CT measurements of the cardiac axis radius (CARD AXIS) from the day of admission (CT CARD AXIS ADM), the fourth day of treatment (CT CARD AXIS D04) and the day of discharge of each patient (CT CARD AXIS DIS) and previous CT cardiac axis radius measurements from clinical records for some of the patients (CT CARD AXIS PRE).

2. Statistical model

To analyze the relationship between the variation of the P_{aO2}/F_{IO2} ratio and the NEG BAL variables we used a linear regression model of the form

$$\text{PAFI BAL} \sim \beta_0 + \beta_1 \text{NEG BAL} \quad (1)$$

For the model (1), the response variable PAFI BAL was computed as the difference between the $P_{aO}2$ /$F_{IO}2$ ratio at the moment of admission and the $P_{aO}2$ /$F_{IO}2$ ratio at the moment of discharge. A paired comparison test was also performed between PAFI ADM and PAFI D04, PAFI ADM and PAFI D08 and PAFI ADM and PAFI DIS and between PAFI ADM and PAFI D04, PAFI D04 and PAFI D08 and PAFI D08 and PAFI DIS.

For the HCT variable, a paired difference test was performed and the corresponding confidence interval was computed for the sample mean of the differences between HCT PRE and HCT ADM and HCT ADM and HCT DIS. This accounts for the statistical significance in the difference of the means of the HCT variable. Analogously, regarding the CT score data, a paired difference test was performed for CT SCORE PRE and CT SCORE ADM, CT SCORE ADM and CT SCORE D04, and CT SCORE D04 and CT SCORE D08. The same test was also performed CT Ø SCV PRE and CT Ø SVC ADM, CT Ø SVC ADM and CT Ø SVC D04 and for CT Ø SVC ADM and CT SVC DIS, and for the corresponding data of the cardiac axis radius.

3. Results

---

[1] The length of the treatment depended on the evolution of each patient.

Table 1 shows the data for Negative Balance and PAFI BAL, and Table 2 the square meters and the normalized NEG BAL (NORM NEG BAL). We detected some outliers, or atypical points, (most notably patient 10) and discarded the possibility of them being influential points via an analysis of residuals vs leverage. Since they do not affect the significance of the model we decided to keep it.

For the model (1) the *p* value obtained is 0.03482. Furthermore, for the data PAFI ADM, PAFI D04, PAFI D08 and PAFI DIS corresponding data in Table 3. Table 4 displays the results of the paired comparison test between PAFI ADM and PAFI D04, PAFI ADM and PAFI D08 and PAFI ADM and PAFI DIS; the mean of the differences are, respectively, -128.65, -201.1579 and -214.3. The corresponding results for comparison between PAFI ADM and PAFI D04, PAFI D04 and PAFI D08 and PAFI D08 and PAFI DIS are detailed in Table 5; the respective mean of the differences are -128.65, -76.94737 and -214.3. The total Negative Balance (of all patients) reported is 152746; the total *m2* is 38.65 and the average is 3952 ml/*m2*.

Table 1. Negative Balance and PAFI BAL data.

CASE	***NEG BAL Accumulated***	***PAFI BAL***
**1**	-3425	104
**2**	-11250	250
**3**	-8800	137
**4**	-6937	264
**5**	-9350	162
**6**	-10250	247
**7**	-4600	194
**8**	-10880	290
**9**	-6800	223
**10**	-7630	25
**11**	-3647	264
**12**	-9439	217
**13**	-7838	237
**14**	-7458	129
**15**	-5645	117
**16**	-5490	163
**17**	-3298	32
**18**	-9160	73
**19**	-11601	395
**20**	-9248	199
**Mean**	-7637	186.1
**Median**	-7734	196.5
**Standard deviation**	-2616.88	91.85

PAFI: $P_{aO}2$ /$F_{IO}2$ ratio, NEG BAL Accumulated: Negative Fluid Balance Accumulated in milliliters total days

Table 2. Body surface in Square Meters and NORM NEG BAL data.		
CASE	m^2	**NORM NEG BAL**
**1**	1.97	-1738.58
**2**	1.81	-6215.47
**3**	1.78	-4943.82
**4**	1.75	-3964
**5**	2.39	-3912.13
**6**	1.71	-5994.15
**7**	1.71	-2690.1
**8**	2.34	-4649.57
**9**	2.25	-3022.22
**10**	2.01	-3796.02
**11**	1.80	-2026.11
**12**	2.13	-4431.45
**13**	1.91	-4103.66
**14**	2.00	-3729
**15**	2.00	-2822.5
**16**	1.84	-2983.7
**17**	1.42	-2322.53
**18**	2.03	-4512.31
**19**	1.65	-7030.91
**20**	2.15	-4301.39
**Mean**	1.93	-3959
**Median**	1.94	-3938
**Standard deviation**	0.24	-1394.37

Table 3. $P_{aO}2$ /$F_{IO}2$ ratio on the day of admission, the fourth, the eight and the discharge days.

CASE	***PAFI ADM***	***PAFI Day 4***	***PAFI Day 8***	***PAFI DIS***
**1**	60	222	195	-
**2**	62	247	366	463
**3**	65	142	340	309
**4**	188	460	-347	
**5**	56	72	218	323
**6**	110	328	-357	
**7**	61	130	255	342
**8**	100	130	390	300
**9**	177	400	-400	
**10**	86	111	114	184
**11**	120	205	404	404
**12**	121	233	323	352
**13**	101	253	380	380
**14**	185	314	-314	
**15**	145	268	341	341
**16**	197	360	-319	
**17**	102	105	225	-
**18**	160	190	233	233
**19**	104	376	499	499
**20**	162	375	-361	
**Mean**	118.10	246.8	316.9	332.4
**Median**	107	240	340	341.5
**Standard deviation**	47.08	111.45	90.49	80.62

PAFI: $P_{aO}2$ /$F_{IO}2$ ratio, ADM: Admission, DIS: Discharge

Table 4. Results for the paired differences between PAFI ADM and PAFI Day 4, PAFI ADM and PAFI Day 8 and PAFI ADM and PAFI DIS.

	t	***DF***	***p-value***	***95% confidence interval***
**PAFI ADM and PAFI Day 4**	-6.8121	19	0.000001671	[-168.17765, -89.12235]
**PAFI ADM and PAFI Day 8**	-9.6677	19	0.00000001498	[-244.8721, -157.4437]
**PAFI ADM and PAFI DIS**	-10.651	13	0.000000001887	[-256.4115, -172.1885]

ADM: Admission, DIS: Discharge, PAFI: $P_{aO}2$ /$F_{IO}2$ ratio

Table 5. Results for the paired differences between PAFI ADM and PAFI Day 4, PAFI Day 4 and PAFI Day 8 and PAFI Day 8 and PAFI DIS.

	t	*DF*	*p-value*	*95% confidence interval*
**PAFI ADM and PAFI Day 4**	-6.8121	19	0.000001671	[-168.17765, -89.12235]
**PAFI Day 4 and PAFI Day 8**	-3.535	18	0.002366	[-122.67906, -31.21567]
**PAFI Day 8 and PAFI DIS**	-1.3089	18	0.207	[-36.334428, 8.439691]

ADM: Admission, DIS: Discharge, PAFI: $P_{aO}2$ /$F_{IO}2$ ratio

Days of admission and discharge, as well as the previous available data, for HCT can be found in Table 6. The results of the paired difference tests are in Table 7; the mean of the differences between HCT PRE and HCT ADM and HCT ADM and HCT DIS (respectively) is 5.707143 and 3.215.

Table 6. HCT PRE, HCT ADM and HCT DIS data.

CASE	*HCT PRE*	*HCT ADM*	*HCT DIS*
**1**	46	43.1	43.5
**2**	28.1	25	31
**3**	41.2	37	44.9
**4**	38	29.3	44.6
**5**	-	42.7	42.4
**6**	-	43	43
**7**	40.1	35	34.4
**8**	-	41	44.7
**9**	41.4	38	39.6
**10**	41	37.4	40.8
**11**	-	38	41.3
**12**	39.6	35.3	38
**13**	-	45	43.7
**14**	-	39	43
**15**	49	35.7	35
**16**	49.9	41	51.6
**17**	39.9	38.3	39
**18**	38.2	34.9	37
**19**	40	31.3	31.8
**20**	49	40	45
**Mean**	41.51	37.5	40.72
**Median**	40.55	38	41.85
**Standard deviation**	5.67	4.93	5.06

HCT: Hematocrit, PRE: Previous, ADM: Admission, DIS: Discharge

Table 7. Results for the paired differences between HCT PRE and HCT ADM and HCT ADM and HCT DIS.

	t	*DF*	*p-value*	*95% confidence interval*
**HCT PRE and HCT ADM**	6.3467	13	0.00002549	[3.764465, 7.649821]
**HCT ADM and HCT DIS**	-3.4341	19	0.002782	[-5.174509, -1.255491]

HCT: Hematocrit, PRE: Previous, ADM: Admission, DIS: Discharge

For the CT score, we have the data in Table 8. The outlier in CT DIS does not affect the significance of the results. The results for the paired difference tests are in Table 9; the mean of the differences between CT SCORE PRE and CT SCORE ADM 10.16667, between CT SCORE ADM and CT SCORE D04 6.55 and between CT SCORE D04 and CT SCORE D08 2.9.

Table 8. CT Score PRE, CT Score ADM, CT Score Day 4 and CT Score Day 8 data.

CASE	*CT Score PRE*	*CT Score ADM*	*CT Score Day 4*	*CT Score Day 8*
**1**	6	21	14	7
**2**	-	13	8	4
**3**	12	20	3	3
**4**	5	17	1	1
**5**	10	17	17	7
**6**	7	14	10	10
**7**	10	23	23	16
**8**	8	19	5	5
**9**	9	18	12	12
**10**	-	25	25	25
**11**	-	12	2	0
**12**	10	20	10	3
**13**	2	16	9	5
**14**	6	11	3	3
**15**	-	22	18	12
**16**	1	12	10	9
**17**	-	17	12	7
**18**	-	17	17	16
**19**	-	11	6	5
**20**	-	19	8	5
**Mean**	7.16	17.2	10.6	7.75
**Median**	7.5	17	10	6
**Standard deviation**	1.43	1.34	1.62	1.41

CT Score: Computed Tomography Score, PRE: Previous, ADM: Admission

Table 9. Results for the paired differences between CT SCORE ADM and CT SCORE Day 4 and CT SCORE Day 4 and CT SCORE Day 8.

	t	*DF*	*p-value*	*95% confidence interval*
**CT SCORE PRE and CT SCORE ADM**	-11.472	11	0.00001845	[-12.11718, -8.21615]
**CT SCORE ADM and CT SCORE Day 4**	5.6376	19	0.00001.954	[4.118244, 8.981756]
**CT SCORE Day 4 and CT SCORE Day 8**	4.1252	19	0.0005757	[1.428602, 4.371398]

CT Score: Computed Tomography Score, PRE: Previous, ADM: Admission

For the CT superior vena cava, we have the data in Table 10. A boxplot CT Ø SVC ADM and CT Ø SVC DIS is shown in Figure 1. The results for the paired difference tests are in Tables 11 and 12; the mean of the differences between CT Ø SVC PRE and CT Ø SVC ADM -4.581818, between CT Ø SVC ADM and CT Ø SVC D04 is 3.631 and between CT Ø SVC ADM and CT Ø SVC DIS is 4.02.

Table 10. CT Ø SVC PRE, CT Ø SVC ADM, CT Ø SVC Day 4 and CT Ø SVC DIS data.

CASE	*CT Ø SVC PRE*	*CT Ø SVC ADM*	*CT Ø SVC Day 4*	*CT Ø SVC DIS*
**1**	17.1	19.8	17.8	16.8
**2**	-	19.1	16.4	15
**3**	18.4	25.2	12.4	12.4
**4**	12.5	16.7	11.4	11.4
**5**	15.5	18.3	17.4	15.9
**6**	13.9	20.1	15.5	15.5
**7**	22.7	25.4	26.5	17.2
**8**	8.3	20.2	13.2	13.2
**9**	19.2	20.9	19.4	19.4
**10**	-	18.3	19.4	17.4
**11**	-	12.3	5.5	8.1
**12**	12.8	19.5	13.6	12.5
**13**	10.7	14.5	10.8	8.1
**14**	-	14.4	12.2	12.2
**15**	-	15.9	15.1	15
**16**	9.5	10.4	13.9	12.6
**17**	-	17.1	10.58	14.4
**18**	-	16.1	15.5	15.5
**19**	-	20	9.1	15.9
**20**	-	14.2	10.1	9.5
**Mean**	14.6	17.92	14.29	13.9
**Median**	13.9	18.3	13.75	14.7
**Standard deviation**	4.44	3.81	4.57	3.08

CT: Computed Tomography, PRE: Previous, ADM: Admission, DIS: Discharge Ø SVC: Diameter Superior Vena Cava

Table 11. Results for the paired differences test between CT Ø SVC PRE and CT Ø SVC ADM and CT Ø SVC ADM and CT Ø SVC Day 4.

	t	*DF*	*p-value*	*95% confidence interval*
**CT Ø SVC PRE and CT Ø SVC ADM**	-4.843	10	0.0006785	[-6.689805, -2.473832]
**CT Ø SVC ADM and CT ØSVC Day 4**	4.0454	19	0.0006907	[1.752373, 5.509627]

CT: Computed Tomography, PRE: Previous, ADM: Admission, Ø SVC: Diameter Superior Vena Cava

Table 12. Results for the paired differences *t*−test between CT Ø SVC ADM and CT Ø SVC DIS.

	t	*DF*	*p-value*	*95% confidence interval*
**CT Ø SVC ADM and CT SVC DIS**	5.468	19	0.00002826	[2.481228, 5.558772]

CT: Computed Tomography, ADM: Admission, DIS: Discharge, Ø SVC: Diameter Superior Vena Cava

Figure 1: Boxplot for CT Ø SVC ADM and CT Ø SVC DIS.

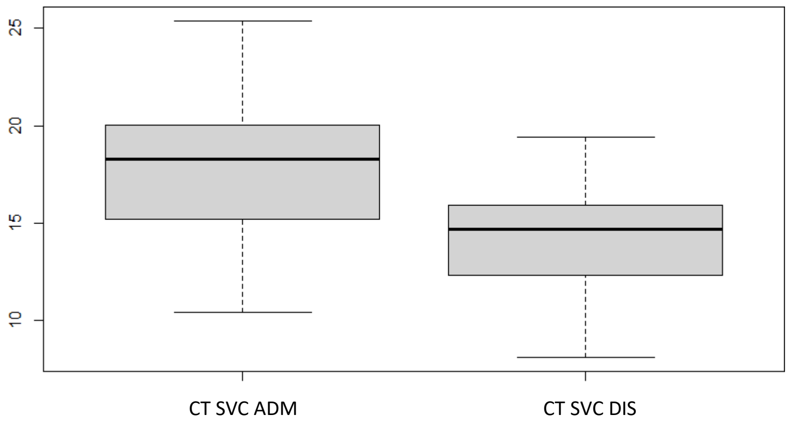

CT: Computed Tomography, ADM: Admission, DIS: Discharge
SVC: Diameter Superior Vena Cava

For the CT cardiac axis radius, we have the data in Table 13. A boxplot for CT CARD AXIS ADM and CT CARD AXIS DIS is shown in Figure 2. The results for the paired difference tests are in Tables 14 and 15; the mean of the differences between CT CARD AXIS PRE and CT CARD AXIS ADM -

1.016667, between CT CARD AXIS ADM and CT CARD AXIS D04 is 1.125 and between CT CARD AXIS ADM and CT CARD AXIS DIS is 1.16.

Table 13. CT CARD AXIS PRE, CT CARD AXIS ADM, CT CARD AXIS Day 4 and CT CARD AXIS DIS data.

CASE	*CT CARD AXIS PRE*	*CT CARD AXIS ADM*	*CT CARD AXIS Day 4*	*CT CARD AXIS DIS*
**1**	11.7	12.5	12.4	12.3
**2**	-	12.8	10.8	11.3
**3**	12.2	14	11.8	11.8
**4**	10.1	11.2	8.3	8.3
**5**	13.7	14.7	14.4	13.5
**6**	10.5	11.8	9.3	9.3
**7**	13.1	13.4	13.9	12.7
**8**	10.9	13.6	11.4	11.4
**9**	14	15	12.9	12.9
**10**	-	12	10.7	10.9
**11**	-	11.4	10	10
**12**	14.2	15	14.1	14.1
**13**	11.7	13.8	13.4	13.1
**14**	13.4	12.6	11.4	11.4
**15**	-	11.5	10.6	10.9
**16**	11	11.1	12	11.7
**17**	-	11.7	10.8	12.1
**18**	-	12.5	12	12
**19**	-	11.3	10.7	10.9
**20**	-	14.5	13	12.6
**Mean**	12.21	12.82	11.7	11.66
**Median**	11.95	12.55	11.6	11.75
**Standard deviation**	1.43	1.34	1.62	1.4

CT: Computed Tomography, PRE: Previous, ADM: Admission, DIS: Discharge, CARD: Cardiac

Table 14. Results for the paired differences test between CT CARD AXIS PRE and CT CARD AXIS ADM and CT CARD AXIS ADM and CT CARD AXIS Day 4.

	t	*DF*	*p-value*	*95% confidence interval*
**CT CARD AXIS PRE and CT CARD AXIS ADM**	-3.7984	11	0.002952	[-1.6057805, -0.4275529]
**CT CARD AXIS ADM and CT CARD AXIS Day 4**	5.013	19	0.00007722	[0.6552952, 1.5947048]

CT: Computed Tomography, PRE: Previous, ADM: Admission, DIS: Discharge, CARD: Cardiac

Table 15. Results for the paired differences test between CT CARD AXIS ADM and CT CARD AXIS DIS.

	t	*DF*	*p-value*	*95% confidence interval*
**CT CARD AXIS ADM and CT CARD AXIS DIS**	5.5058	19	0.00002602	[0.7190247, 1.6009753]

CT: Computed Tomography, CARD: Cardiac, ADM: Admission, DIS: Discharge

Figure 2: Boxplot for CT CARD AXIS ADM and CT CARD AXIS DIS.

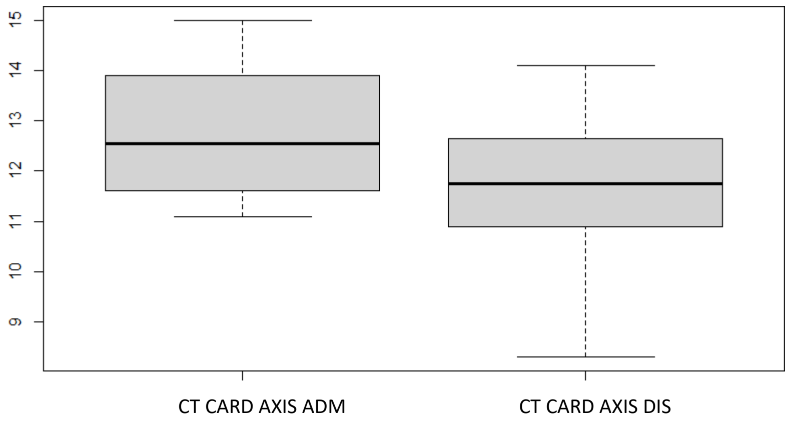

CT: Computed Tomography, ADM: Admission, DIS: Discharge, CARD: Cardiac

4. Appendix: security variables

In this appendix we gather the statistical analyses regarding the security variables; the data for these analyses comprise: measurements of the patients' creatinine levels at the moment of admission and discharge (CREAT ADM and CREAT DIS, see Table 16), and sodium and potassium levels at the moment of admission and the seventh day of treatment (Na ADM, Na D07, K ADM and K D07 respectively, presented in Tables 17 and 18), and measurements of the patients' temperature and heart frequency on each day of the treatment, displayed in Tables 19 and 20. Figure 3 shows the evolution of the mean temperature of the patients.

The results for the paired tests for CREAT ADM and CREAT DIS, Na ADM and Na D07 and K ADM and K D07 can be found in Tables 21, 22 and 23, and the respective boxplots in Figures 4, 5 and 6. Further, Table 24 shows the minimum and maximum values, mean, median and quartiles for each of these variables.

Figure 3: Evolution of the mean temperature.

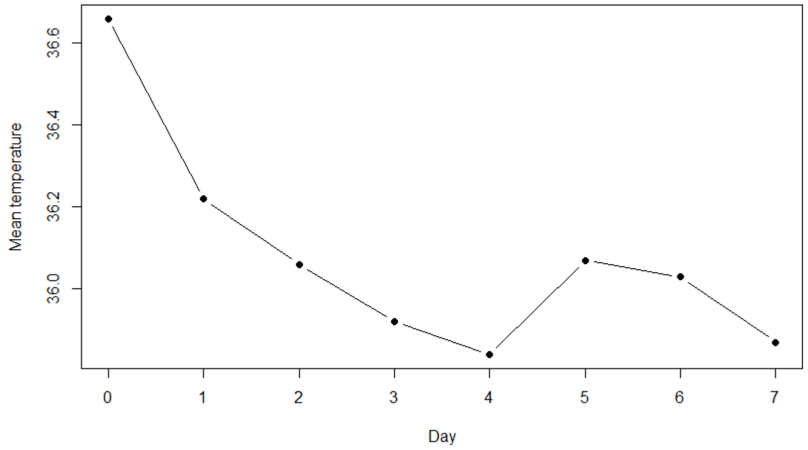

Figure 4: Boxplot for CREAT ADM and CREAT DIS.

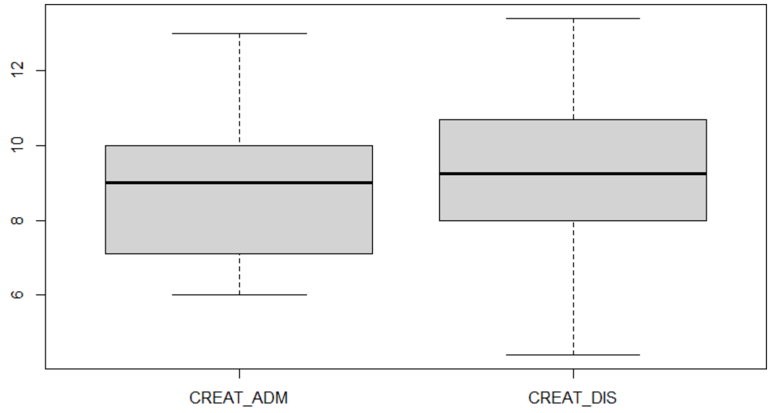

CREAT: Serum Creatinine, ADM: Admission, DIS: Discharge

Table 21. Results for the paired differences test between CREAT ADM and CREAT DIS

	t	*DF*	*p-value*	*95% confidence interval*
**CREAT ADM and CREAT DIS**	-1.307	17	0.2086	[-1.5394756, 0.3616978]

CREAT: Serum Creatinine, ADM: Admission, DIS: Discharge

Table 22. Results for the paired differences test between Na ADM and Na DIS

	t	*DF*	*p-value*	*95% confidence interval*
**Na ADM and Na DIS**	2.3353	11	0.0395	[0.1102342, 3.7230992]

Na: Sodium, ADM: Admission, DIS: Discharge

Figure 5: Boxplot for Na ADM and Na DIS.

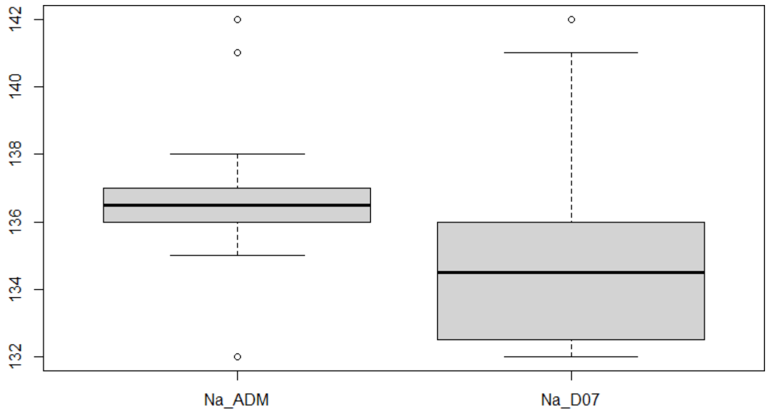

Na: Sodium, ADM: Admission, DIS: Discharge

Figure 6: Boxplot for K ADM and K Day 7.

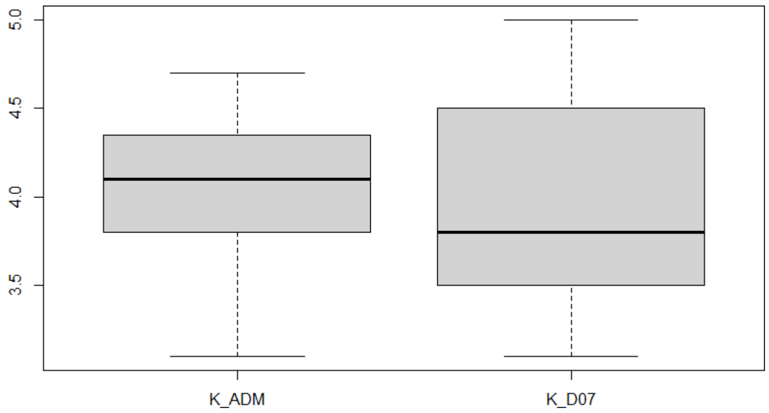

K: Potassium, ADM: Admission, D07: Day 07

Table 23. Results for the paired differences test between K ADM and K Day 7

	t	*DF*	*p-value*	*95% confidence interval*
**K ADM and K Day 7**	0.095218	12	0.9257	[-0.5049770, 0.5511309]

K: Potassium, ADM: Admission

Table 24. Minimum, maximum, quartiles, median and mean for CREAT ADM, CREAT DIS, Na ADM, Na Day 7, K ADM and K Day 7.

	Min	*1st Q*	*Median*	*Mean*	*3rd Q*	*Max*
**CREAT ADM**	6	7.1	9	8.865	10	13
**CREAT DIS**	4.4	8.075	9.250	9.328	10.625	13.4
**Na ADM**	132	136	136.5	136.8	137	142
**Na Day 7**	132	132.8	134.5	135.2	135.5	142
**K ADM**	3.1	3.8	4.1	4.025	4.325	4.7
**K Day 7**	3.1	3.5	3.8	3.992	4.5	5

CREAT: Serum Creatinine, Na: Sodium, K: Potassium, ADM: Admission, DIS: Discharge

5. Appendix 2: Cardiothoracic Ratio and furosemide

For the CT Cardiothoracic Ratio (CTR), we have the data in Table 25. A boxplot for CT CTR PRE, CT CTR ADM and CT CTR D04 is shown in Figure 7 and another one for CT CTR ADM and CT CTR D08 is shown in Figure 8. The results for the paired difference tests are in Tables 26 and 27; the mean of the differences between CT CTR PRE and CT CTR ADM -0.05083333, between CT CTR ADM and CT CTR D04 is 0.04789474 and between CT CTR ADM and CT CTR D08 is 0.02333333.

Table 25. CT CTR PRE, CT CTR ADM, CT CTR Day 4 and CT CTR Day 8 data.

CASE	*CT CTR PRE*	*CT CTR ADM*	*CT CTR Day 4*	*CT CTR Day 8*
**1**	0.45	0.47	-	0.45
**2**	-	0.56	0.48	-
**3**	0.45	0.54	0.45	-
**4**	0.39	0.44	0.34	-
**5**	0.44	0.47	0.47	0.44
**6**	0.40	0.49	0.37	-
**7**	0.55	0.57	0.60	0.55
**8**	0.37	0.52	0.41	-
**9**	0.47	0.51	0.44	-
**10**	-	0.47	0.42	0.47
**11**	-	0.50	0.43	0.44
**12**	0.52	0.57	0.53	0.51
**13**	0.42	0.49	0.48	0.47

**14**	0.48	0.46	0.41	-
**15**		0.44	0.40	0.42
**16**	0.42	0.44	0.48	0.46
**17**	-	0.53	0.48	0.55
**18**	-	0.51	0.48	-
**19**	-	0.47	0.45	0.45
**20**	-	0.54	0.49	0.47
**Mean**	0.45	0.49	0.45	0.47
**Median**	0.44	0.495	0.45	0.46
**Standard deviation**	0.05	0.04	0.06	0.04

PRE: Previous, ADM: Admission, CT: Computed Tomography, CTR: Cardiothoracic Ratio

Table 26. Results for the paired differences test between CT CTR PRE and CT CTR ADM and CT CTR ADM and CT CTR Day 4.

	t	*DF*	*p-value*	*95% confidence interval*
**TAC CTR PRE and TAC CTR ADM**	-3.9654	11	0.002213	[-0.07904811, -0.02261856]
**TAC CTR ADM and TAC CTR Day 4**	4.7814	18	0.0001493	[0.02684998, 0.06893949]

PRE: Previous, ADM: Admission, CT: Computed Tomography, CTR: Cardiothoracic Ratio

Table 27. Results for the paired differences test between CT CTR ADM and CT CTR Day 8.

	t	*DF*	*p-value*	*95% confidence interval*
**CTR ADM and CTR Day 8**	2.7849	11	0.01775	[0.004892017, 0.041774650]

ADM: Admission, CT: Computed Tomography, CTR: Cardiothoracic Ratio

Figure 7: Boxplot for CT CTR PRE, CT CTR ADM and CT CTR Day 4.

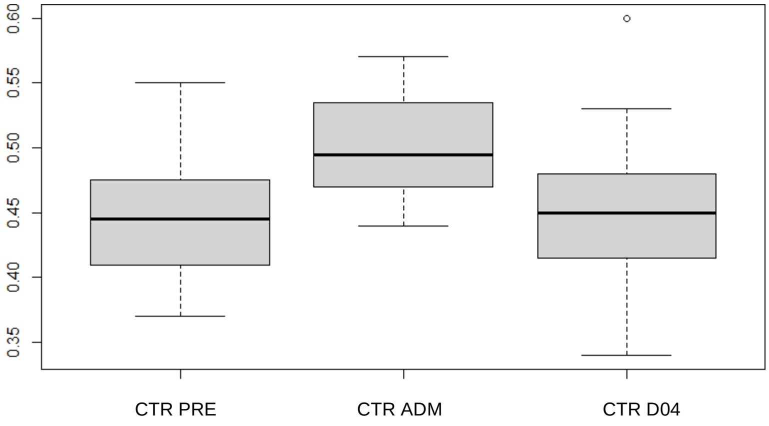

ADM: Admission, CT: Computed Tomography, CTR: Cardiothoracic Ratio, D04: Day 4

Figure 8: Boxplot for CT CTR ADM, CT CTR Day8.

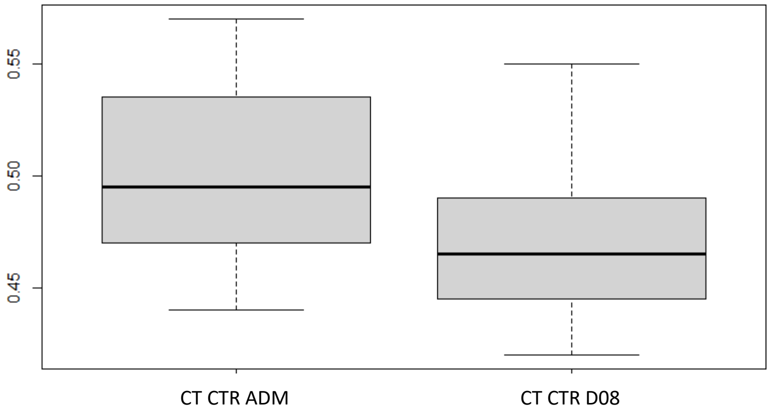

ADM: Admission, CT: Computed Tomography, CTR: Cardiothoracic Ratio, D08: Day 8
